# Kidney function trajectories before and after hospitalization for heart failure with reduced ejection fraction

**DOI:** 10.1093/eurheartj/ehaf457

**Published:** 2025-07-30

**Authors:** Masatake Kobayashi, Antoni Bayes-Genis, Kevin Duarte, John J V McMurray, João Pedro Ferreira, Stuart J Pocock, Dirk J Van Veldhuisen, Josep Lupón, Bertram Pitt, Faiez Zannad, Nicolas Girerd

**Affiliations:** Université de Lorraine, and the CardioRenal Integration Clinical Investigation Center, INSERM, CHU de Nancy, Rue du Morvan, 54500 Vandoeuvre-les-Nancy, France; Department of Cardiology, Tokyo Medical University, Tokyo, Japan; Heart Institute, University Hospital Germans Trias i Pujol, Departament de Medicina, Universitat Autònoma de Barcelona, Barcelona, Spain; Université de Lorraine, and the CardioRenal Integration Clinical Investigation Center, INSERM, CHU de Nancy, Rue du Morvan, 54500 Vandoeuvre-les-Nancy, France; BHF Cardiovascular Research Centre, Institute of Cardiovascular and Medical Sciences, University of Glasgow, Glasgow, UK; Université de Lorraine, and the CardioRenal Integration Clinical Investigation Center, INSERM, CHU de Nancy, Rue du Morvan, 54500 Vandoeuvre-les-Nancy, France; Cardiovascular Research and Development Center, Department of Surgery and Physiology, Faculty of Medicine of the University of Porto, Porto, Portugal; Department of Medical Statistics, London School of Hygiene and Tropical Medicine, London, UK; Department of Cardiology, University of Groningen, Groningen, The Netherlands; Heart Institute, University Hospital Germans Trias i Pujol, Departament de Medicina, Universitat Autònoma de Barcelona, Barcelona, Spain; Department of Medicine, University of Michigan School of Medicine, Ann Arbor, MI, USA; Université de Lorraine, and the CardioRenal Integration Clinical Investigation Center, INSERM, CHU de Nancy, Rue du Morvan, 54500 Vandoeuvre-les-Nancy, France; Université de Lorraine, and the CardioRenal Integration Clinical Investigation Center, INSERM, CHU de Nancy, Rue du Morvan, 54500 Vandoeuvre-les-Nancy, France

**Keywords:** heart failure with reduced ejection fraction, Kidney function, Cardiorenal syndrome, Mineralocorticoid receptor antagonist, Heart failure hospitalization

## Abstract

**Background and Aims:**

Worsening kidney function is a key prognostic factor in heart failure (HF) with reduced ejection fraction (HFrEF). However, associations between kidney function trajectories and HF-related events remain unclear.

**Methods:**

Longitudinal changes in estimated glomerular filtration rate (eGFR) before and after a HF-related event, defined as HF hospitalization or HF death, were examined using individual patient data from two clinical trials (EPHESUS and EMPHASIS-HF) and a real-world cohort (BARCELONA).

**Results:**

HF-related events occurred in 14.1% of 8587 patients [EPHESUS/EMPHASIS-HF; median follow-up 17.1 (12.4–22.7) months] and 33.8% of 2048 patients [BARCELONA; median 47.0 (18.8–90.6) months]. In EPHESUS and EMPHASIS-HF, patients who experienced an HF-related event had a steeper decline in eGFR in the year preceding the event (average −4.83 mL/min/1.73 m²/year) compared with those who did not have an HF-related event (−1.18 mL/min/1.73 m²/year). Over the 1 year following an HF-related event, eGFR continued to decline, though at a slower rate (average −3.45 mL/min/1.73 m²/year). Similar kidney function trajectories were observed in BARCELONA (average eGFR decline −1.35 mL/min/1.73 m²/year in patients without HF event vs −5.77 mL/min/1.73 m²/year 1 year before an event and −3.04 mL/min/1.73 m²/year over the year after an event). Worsening New York Heart Association class paralleled steeper eGFR decline prior to HF events.

**Conclusions:**

In HFrEF, kidney function decline may precede a HF hospitalization or death by up to 1 year, linking to symptomatic congestion. Monitoring eGFR slopes rather than relying solely on specific cut-off values may allow early detection of at-risk patients.

## Structured Graphical Abstract

**Structured Graphical Abstract:**
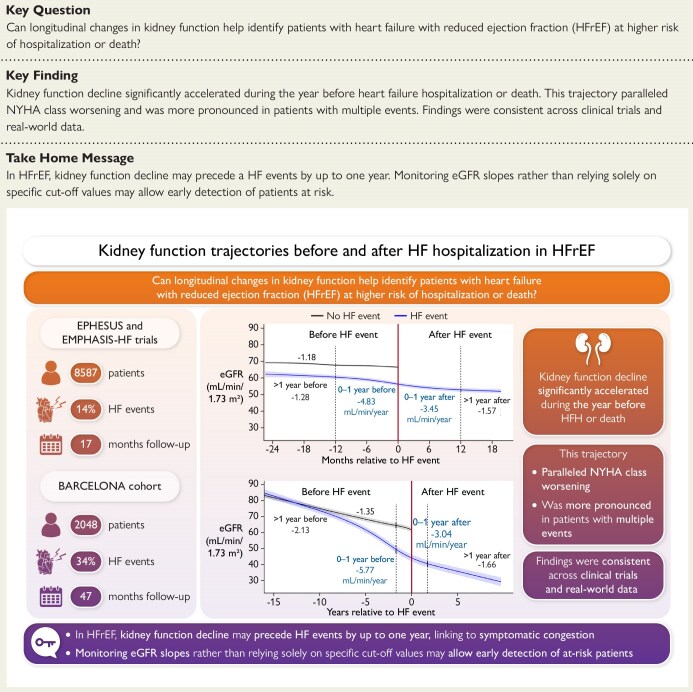
Kidney function trajectories before and after heart failure hospitalization in heart failure with reduced ejection fraction. eGFR, estimated glomerular filtration rate; HF, heart failure; HFH, heart failure hospitalization; HFrEF, heart failure with reduced ejection fraction; NYHA, New York Heart Association.

**See the editorial comment for this article ‘Glomerular filtration rate slope in heart failure: ready for prime time?', by B.L. Neuen and M. Vaduganathan, https://doi.org/10.1093/eurheartj/ehaf643.**

## Introduction

Patients with heart failure (HF) may experience an urgent hospitalization due to worsening symptoms, or death, despite clinically stabilized symptoms on guideline-directed medical therapy.^1^ Hospitalization due to worsening HF is associated with a higher risk of subsequent readmission or death, particularly in patients with HF and reduced ejection fraction (HFrEF).^2–4^

Kidney disease is common in patients with HF, and kidney function declines progressively in HF, compared with other cardiovascular (CV) diseases.^5^ There is also compelling evidence that poor kidney function is associated with a worse prognosis in HFrEF.^6–8^ Furthermore, among patients with HF and preserved ejection fraction (HFpEF) in the Prospective Comparison of ARNI with ARB Global Outcomes in Heart Failure with Preserved Ejection Fraction (PARAGON-HF) trial, steeper rates of decline in kidney function were observed in the year preceding hospitalization in patients admitted due to worsening HF, compared with rate of decline in kidney function among patients not hospitalized for this reason.^9^ However, kidney function trajectories in patients with HFrEF preceding and following HF-related events are uncertain.

We aimed to study the trajectories of kidney function preceding and following HF-related events using individual patient data from two clinical trials: The Eplerenone Post Acute Myocardial Infarction Heart Failure Efficacy and Survival Study (EPHESUS),^10^ and the Eplerenone in Mild Patients Hospitalization and Survival Study in Heart Failure (EMPHASIS-HF),^11^ and ‘real world’ data from the BARCELONA cohort.^12^ Additionally, we assessed whether mineralocorticoid receptor antagonist (MRA) therapy influenced the association between change in kidney function and HF events.

## Methods

### Study populations

In the EPHESUS trial, 6632 patients with a left ventricular ejection fraction (LVEF) ≤ 40% after acute myocardial infarction (MI) who had signs and symptoms of HF or diabetes were randomly allocated to receive either eplerenone or placebo. Key exclusion criteria were estimated glomerular filtration rate (eGFR) < 30 min/min/1.73 m^2^ or serum potassium >5.0 mmol/L at screening as reported elsewhere.^10^

In the EMPHASIS-HF trial, 2737 patients with HF and an LVEF ≤30% (or, if >30 to 35%, a QRS duration of >130 ms on electrocardiography), and New York Heart Association (NYHA) class II were randomly assigned to receive either eplerenone or placebo (ClinicalTrials.gov NCT00232180). Key exclusion criteria were acute MI, NYHA class III or IV, eGFR <30 mL/min/1.73 m^2^ or serum potassium >5.0 mmol/L at screening as reported elsewhere.^11^

In the BARCELONA cohort, ambulatory patients with HF referred to a structured multidisciplinary HF clinic of a university hospital in northern Barcelona between August 2001 and December 2021 were included. HF was defined according to current European Society of Cardiology guidelines at the time of enrolment, and patients with HF and an LVEF ≤40% were analysed. Serum creatinine was measured with the Siemens CREA method (FD33A) on a Dimension® RxL Clinical Chemistry System (Siemens) at the prospectively scheduled timing (i.e. baseline, Month 1 and every 3 months thereafter) and non-scheduled visits (drug titration, outpatient decompensations, etc.).^12,13^

The eGFRs from all studies were recalculated using the Chronic Kidney Disease Epidemiology Collaboration formula.^14^ We included patients with at least two measures of eGFR during follow-up, with exclusion of all eGFR values <10 or >150 mL/min/1.73 m^2^.

Each study was conducted in accordance with the Declaration of Helsinki, and approved by the site ethics committees. All participants gave written informed consent to participate in the respective studies. This secondary analysis was approved by the local ethics committee of Nancy University Hospital.

### Statistical analysis

Continuous variables are presented as mean ± standard deviation or median (25th and 75th percentiles) for data with skewed distributions, and categorical variables as frequency (percentage). Comparisons of baseline characteristics by HF events during the study period were analysed using *t*-test or Wilcoxon test for continuous variables and χ2 or Fisher’s exact test for categorical variables.

An HF event was defined as the composite of HF hospitalization or death from HF progression. Patients were followed during a median period of 17.1 (12.4–22.7) months in the EPHESUS and EMPHASIS-HF trials, and 47.0 (18.8–90.6) months in the BARCELONA cohort.

In each population, the temporal trajectory of eGFR before and after an HF event occurring during follow-up was examined using repeated measures linear mixed-effects regression models, with a random intercept for each patient to account for within-patient correlation. eGFR was the dependent variable, and time relative to the HF event was the explanatory variable. Time was centred on the date of the HF event (*T* = 0) and modelled using linear piecewise splines with predefined knots at −1, 0, and +1 year, estimating eGFR slope across four distinct periods: > 1 year before the HF event, 1 year before the event, 1 year after the event, and >1 year after the event.

To provide a comparison group, we estimated the eGFR slope among patients without HF events using the same model structure, defining time as the number of months before the end of follow-up (i.e. centred on the date of last follow-up) and modelling it as a simple linear function. The eGFR slopes in each period around the HF event were then compared with the slope observed in the reference population who remained free of HF event.

For graphical representations, we used restricted cubic splines to model time separately for each group (HF event/no HF event), allowing flexible visualization of potential non-linear trends in eGFR trajectories.

To assess the interplay between eGFR and NYHA class, we explored eGFR trajectories before/after HF events using the same statistical approach, adjusting for NYHA class which was concurrently assessed with each eGFR measurement. NYHA class trajectories as continuous variables were also assessed using the same statistical approach. Additionally, NYHA class trajectories were compared across eGFR slope tertiles in the year before the HF event by stratifying HF event groups with three slope-based categories: Tertile 1 (steep eGFR decline, < −17.5 mL/min/1.73 m²/year), Tertile 2 (mild decline or stable eGFR, −17.5 to 1.9 mL/min/1.73 m²/year), and Tertile 3 (eGFR increase, > 1.9 mL/min/1.73 m²/year). Slopes were calculated using the last measurement >1 year prior, all values within 1 year before, and the first value after the HF event.

All analyses were performed using R software (the R Foundation for Statistical Computing, Vienna, Austria) version 4.2.1. The two-tailed significance level was set at *P* < .05.

## Results

### Patient characteristics

Overall, patients in the BARCELONA cohort had more eGFR measurements over a longer follow-up period, compared with those in the EPHESUS and EMPHASIS-HF trials (frequency of eGFR measurements per patient, 11 [6–20] in BARCELONA vs 5 [4–6] in EPHESUS and EMPHASIS-HF) (*Table 1*).

**Table 1:** Baseline characteristics by HF events during the follow-up period

	EPHESUS and EMPHASIS-HF			BARCELONA		
	No HF event (*N* = 7375)	HF events (*N* = 1212)	*P*-value	No HF event (*N* = 1356)	HF events (*N* = 692)	*P*-value
Number of eGFR measurements per patient	5 (4–6)	5 (3–6)		10 (5–17)	15 (8–25)	
Time to follow-up per patient, months	17.4 (12.7–22.8)	14.2 (8.1–21.2)		42.3 (15.8–84.5)	60.1 (30.0–102.3)	
Age, years	64.4 ± 10.8	69.1 ± 10.0	**<**.**0001**	64.2 ± 12.5	68.3 ± 10.8	**<.0001**
Female, N (%)	1931 (26.2)	373 (30.8)	.**001**	328 (24.2)	170 (24.6)	.87
Race, N (%)			.12			
White	6581 (89.2)	1053 (86.9)				
Black	94 (1.3)	20 (1.7)	.28	7 (0.5)	3 (0.4)	.99
Asian	251 (3.4)	54 (4.5)				
Other	449 (6.1)	85 (7.0)				
BMI, kg/m²	27.5 ± 4.6	27.2 ± 4.8	.**037**			
Systolic BP, mmHg	120.9 ± 16.6	118.5 ± 16.7	**<**.**0001**			
Diastolic BP, mmHg	73.2 ± 10.5	70.9 ± 11.0	**<**.**0001**			
Heart rate, b.p.m.	73.3 ± 11.7	76.1 ± 12.8	**<**.**0001**			
LVEF, %	31.7 ± 6.4	29.4 ± 6.7	**<**.**0001**	29.2 ± 7.1	28.2 ± 7.5	**.003**
Medical history, *N* (%)						
Previous HF hospitalization	1269 (17.2)	401 (33.1)	**<**.**0001**			
HF duration, years				0.3 (0.1–3.0)	1.0 (0.2–4.9)	**<.0001**
Ischemic heart disease				640 (47.2)	428 (61.8)	**<.0001**
Previous history of MI	2279 (30.9)	562 (46.4)	**<**.**0001**	471 (34.7)	345 (49.9)	**<.0001**
Hypertension	4487 (60.8)	835 (68.9)	**<**.**0001**	795 (58.6)	478 (69.1)	**<.0001**
Diabetes	2201 (29.8)	522 (43.1)	**<**.**0001**	523 (38.6)	361 (52.2)	**<.0001**
Atrial fibrillation	1222 (16.6)	311 (25.7)	**<**.**0001**			
NYHA class			**<**.**0001**			**<.0001**
Class I	1671 (23.0)	162 (13.7)		113 (8.4)	19 (2.7)	
Class II	4772 (65.8)	750 (63.6)		990 (73.2)	441 (63.7)	
Class III	766 (10.6)	229 (19.4)		240 (17.7)	224 (32.4)	
Class IV	42 (0.6)	39 (3.3)		10 (0.7)	8 (1.2)	
eGFR, mL/min/1.73m²	69.1 ± 19.8	60.1 ± 20.0	**<**.**0001**	69.6 ± 26.4	60.7 ± 25.4	**<.0001**
eGFR <60 mL/min/1.73m², N (%)	2523 (34.4)	664 (54.9)	**<**.**0001**	493 (36.4)	358 (51.7)	**<.0001**
Potassium, mmol/L	4.3 ± 0.4	4.3 ± 0.5	.11			
Medications, N (%)						
ACE inhibitor/ARB	6519 (88.5)	1101 (91.0)	.**010**			
Beta-blockers	5863 (79.6)	885 (73.1)	**<**.**0001**			
Eplerenone	3773 (51.2)	537 (44.3)	**<**.**0001**			
Diuretic	4676 (63.5)	1021 (84.4)	**<**.**0001**			

Values are expressed as mean ± SD, *n* (%) or median (25th to 75th percentile).

HF, heart failure; eGFR, estimated glomerular filtration rate; BMI, body mass index; BP, blood pressure; LVEF, left ventricular ejection fraction; MI, myocardial infarction; NYHA, New York Heart Association; ACE inhibitor, angiotensin converting enzyme inhibitor; ARB, angiotensin receptor blocker.

Among 8587 patients (mean age, 65.1 ± 10.8 years; female 26.8%) included in the EPHESUS and EMPHASIS-HF trials, and 2048 patients (mean age, 65.6 ± 12.1 years; female 24.3%) included in the BARCELONA cohort, 14.1% and 33.8% of patients, respectively, experienced HF hospitalization or death from HF progression during the study period.

Patients experiencing HF events were older, and had a higher rate of prior HF admissions, a higher prevalence of CV comorbidities, and lower blood pressure, LVEF, and baseline eGFR, compared with those who did not (all *P*<.05) (*Table 1*). In the EPHESUS and EMPHASIS-HF trials, those with HF events had more severe baseline HF symptoms, more frequent use of diuretics, were less likely to be on baseline beta-blocker therapy, and less likely to be assigned to eplerenone compared with those without HF events (all *P* < .05) (*Table 1*).

### Kidney function decline before and after heart failure events

**Figure 1:**
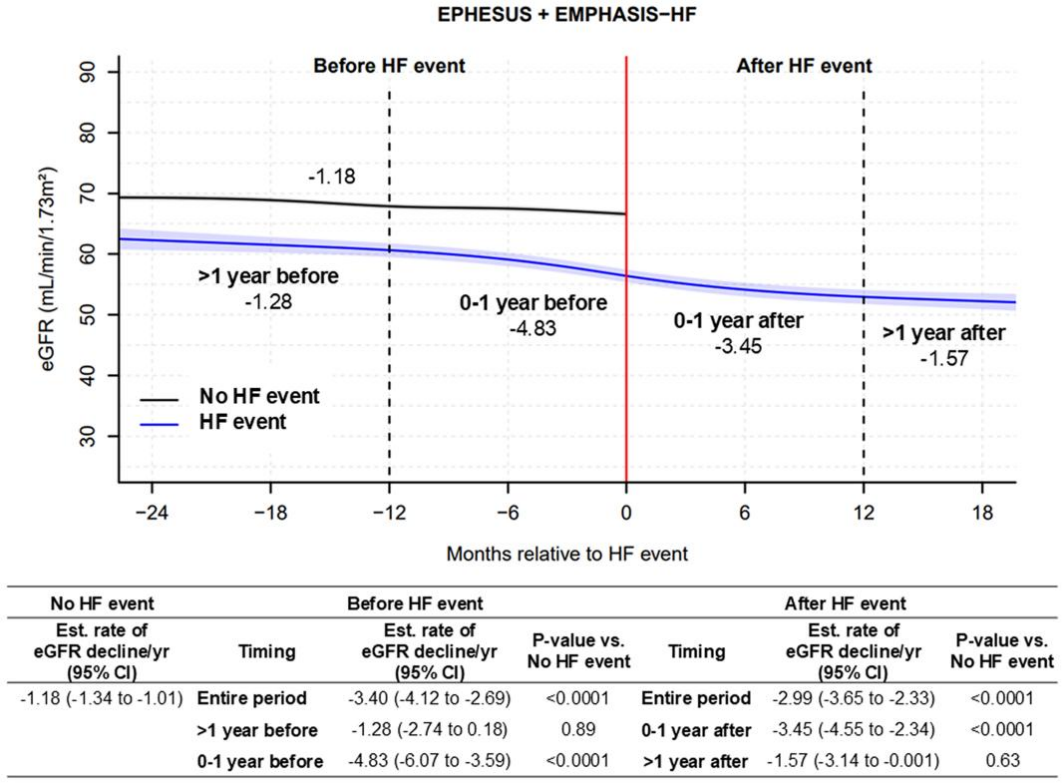
Estimated glomerular filtration rate trajectory before and after heart failure events from the EPHESUS and EMPHASIS-HF trials. HF, heart failure; eGFR, estimated glomerular filtration rate; CI, confidence interval. Number in the figure presents the estimated rate of eGFR decline per year

In the EPHESUS and EMPHASIS-HF trials, median follow-up period before and after HF events was 4.8 (1.4–11.4) months in 14.1% of patients and 8.0 (2.8–14.0) months in 10.8%, respectively. Those with an HF event showed a faster decline in eGFR before experiencing the event compared with those without an HF event [average eGFR decline: −3.40 (−4.12 to −2.69) vs −1.18 (−1.34 to −1.01) mL/min/1.73 m²/year, *P* < .0001] (*Figure 1*). Among patients experiencing an HF event, the rates of eGFR decline were steeper during the year before and after the event, but not over the 1–2 years before or 1–2 years after the event [average eGFR decline: −1.28 (−2.74 to 0.18) mL/min/1.73m²/year >1 year before, −4.83 (−6.07 to −3.59) during 1 year before, −3.45 (−4.55 to −2.34) during 1 year after, and −1.57 (−3.14 to 0.00) > 1 year after an HF event] (*Figure 1*).

As a sensitivity analysis, similar trajectories of kidney function decline before and after HF events were observed only in patients from the EMPHASIS-HF trial (see Supplementary data online, *Table S1*).

**Figure 2:**
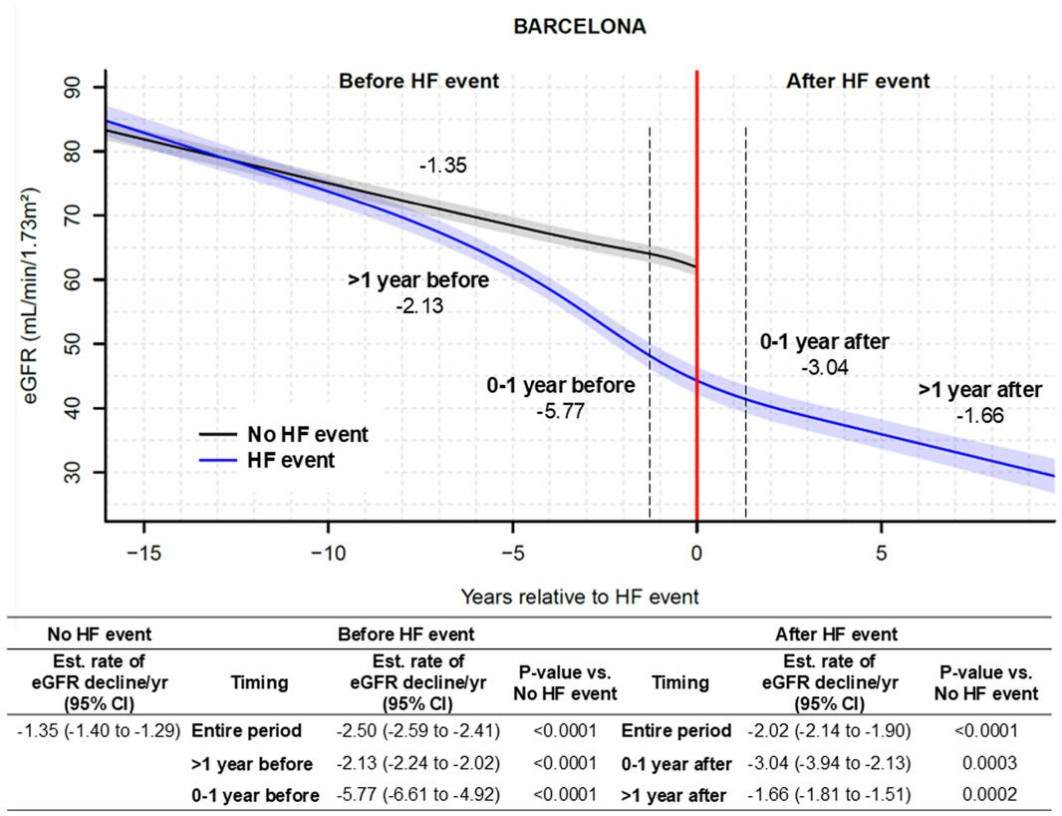
Estimated glomerular filtration rate trajectory before and after heart failure events from the BARCELONA cohort. HF, heart failure; eGFR, estimated glomerular filtration rate; CI, confidence interval. Dotted lines indicate 1 year before and after HF events. Number in the figure presents the estimated rate of estimated glomerular filtration rate decline per year

Comparably, in the BARCELONA cohort, median follow-up period before and after HF events was 26.3 (9.1–62.5) months in 33.8% of patients and 20.7 (7.8–46.4) months in 25.8%, respectively. Those experiencing an HF event had a steeper rate of decline in eGFR before the event compared with those who did not [average eGFR decline: −2.50 (−2.59 to −2.41) vs −1.35 (−1.40 to −1.29) mL/min/1.73 m^2^/year, *P* < .0001] (*Figure 2*). A decline in eGFR was most pronounced over the year before an HF event [average eGFR decline: −5.77 (−6.61 to −4.92) mL/min/1.73 m^2^/year], and this decline persisted over the year after the event [average eGFR decline: −3.04 (−3.94 to −2.13) mL/min/1.73 m^2^/year]. During the second and subsequent years after the event, the rate of decline in eGFR decelerated to approximately the levels observed in patients without HF events, with an average eGFR decline of −1.66 mL/min/1.73 m²/year (*Figure 2*).

Linear representations of the data are presented in Supplementary data online, *Figure S1*.

### Kidney function decline taking into account NYHA

**Figure 3:**
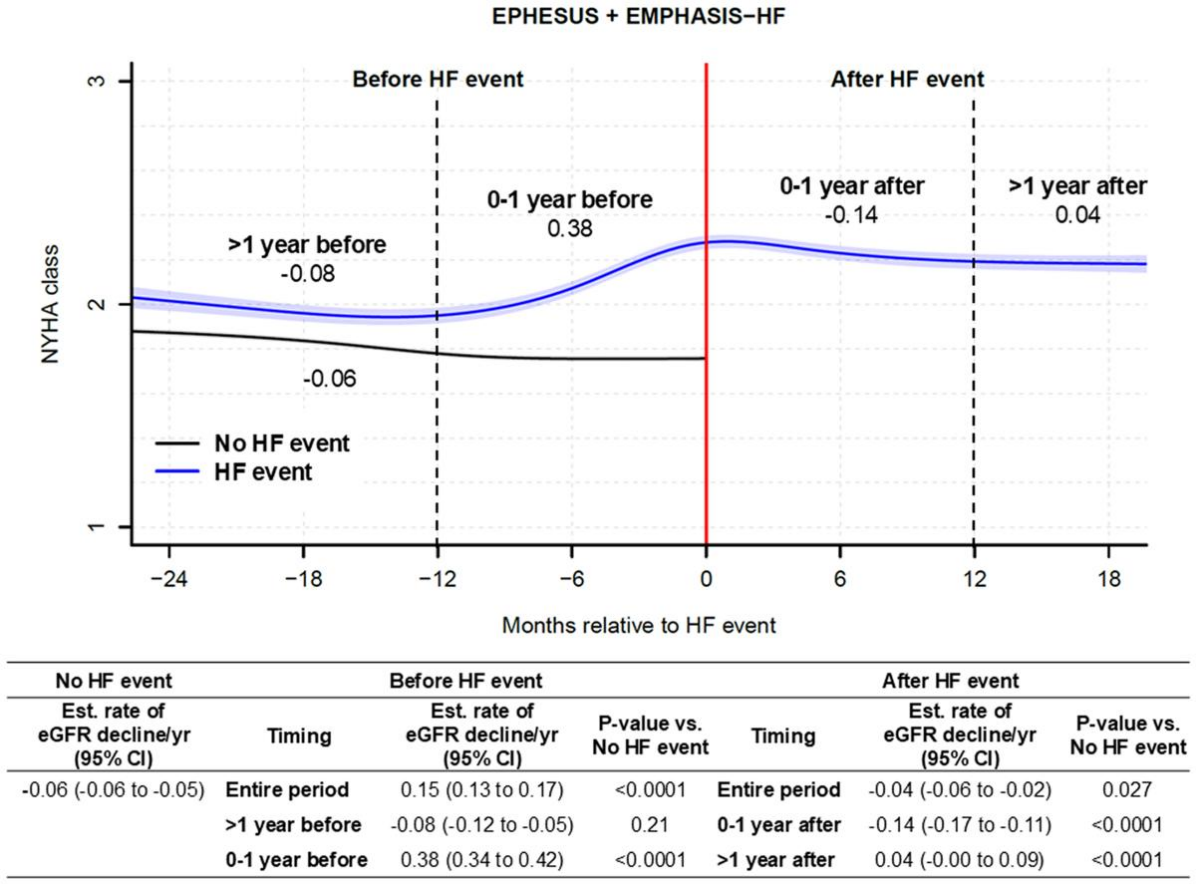
New York Heart Association class trajectory before and after heart failure events from the EPHESUS and EMPHASIS-HF trials. HF, heart failure; NYHA, New York Heart Association; CI, confidence interval. Dotted lines indicate 1 year before and after HF events. Number in the figure presents the estimated rate of estimated glomerular filtration rate decline per year

When adjusting for NYHA class, the eGFR slope in patients with HF events remained steeper compared with those without, although the association was attenuated (see Supplementary data online, *Table S2*). Specifically, during the year before the HF event, the adjusted eGFR slope was −3.31 mL/min/1.73 m²/year in the HF group vs −1.09 mL/min/1.73 m²/year in the no-HF group (*P* = .001). In contrast, adjustment for NYHA did not modify the slope in patients without HF events. Additionally, NYHA class trajectories showed significant worsening beginning 6–9 months before the HF event, consistent with the timing of accelerated eGFR decline (*Figure 3*). Patients in the highest tertile of eGFR slope decline experienced the greatest increase in NYHA class before the event (see Supplementary data online, *Table S3*).

In further analyses (see Supplementary data online, *Table S4*), we found that patients with multiple HF events experienced a steeper and more sustained eGFR decline after the first event, compared with those with a single event. A steeper decline during the year following the first HF event was observed in the EPHESUS and EMPHASIS-HF dataset (−4.37 vs −2.86 mL/min/1.73 m²/year) and in the BARCELONA cohort (−3.73 vs −1.26 mL/min/1.73 m²/year).

### Sensitivity analyses

When restricting the definition of HF event to non-fatal HF hospitalization (i.e. excluding patients whose first event was HF death), the pattern of eGFR decline remained consistent (see Supplementary data online, *Figure S2*). Specifically, the slope of eGFR decline during the year preceding HF hospitalization was −4.64 (−5.90 to −3.38) mL/min/1.73 m²/year in EMPHASIS-HF and EPHESUS dataset, and −5.75 (−6.60 to −4.91) mL/min/1.73 m²/year in BARCELONA cohort (both *P* < .0001) (see Supplementary data online, *Table S5*).

To ensure that post-HF event slopes were not confounded by censoring due to death, we performed an additional analysis considering a non-fatal HF hospitalization group was restricted to patients who survived until the end of follow-up. The results were similar than those observed in previous analyses (see Supplementary data online, *Figure S2* and Supplementary data online, *Table S6*).

When excluding eGFR values within 2 weeks before and after the HF event, results were consistent with the main analysis (see Supplementary data online, *Table S7*).

### Estimated glomerular filtration rate trajectory before and after heart failure events according to randomized treatment assignment (eplerenone/placebo) in EPHESUS and EMPHASIS-HF

**Figure 4:**
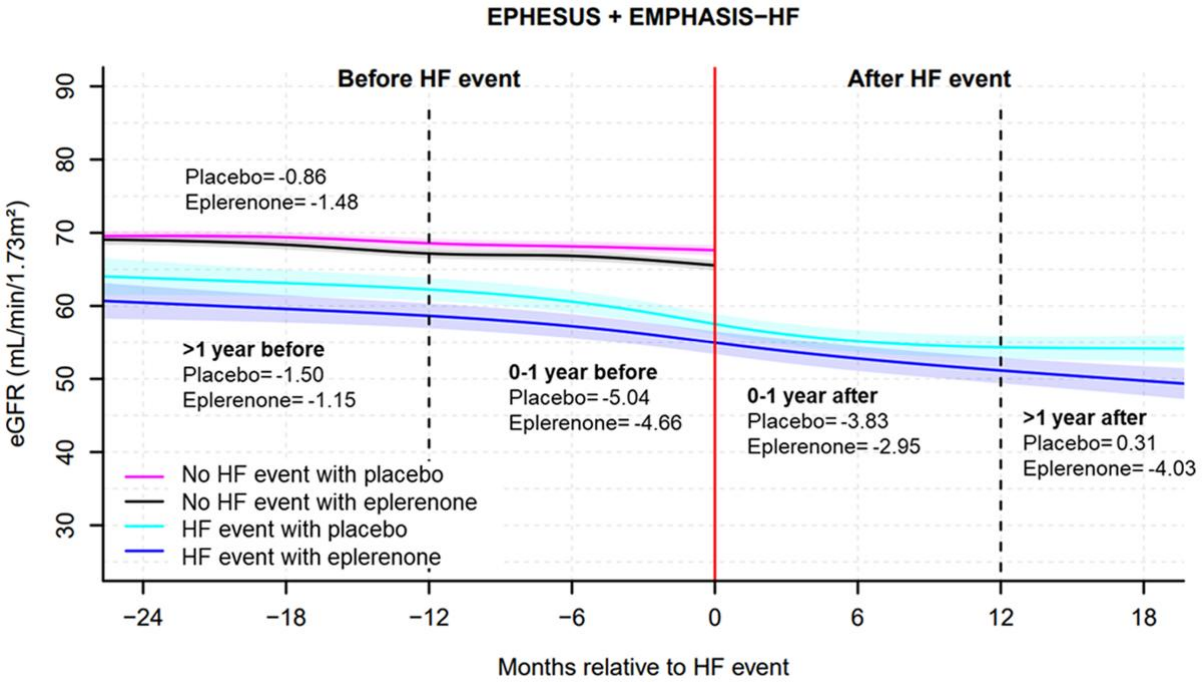
Estimated glomerular filtration rate trajectory before and after heart failure events between eplerenone and placebo groups from the EPHESUS and EMPHASIS-HF trials. HF, heart failure, Number in the figure presents the estimated rate of estimated glomerular filtration rate decline per year in the placebo/eplerenone groups

In the placebo group, patients with an HF event had a steeper decline in eGFR during the year before the event, compared with those without an event [average eGFR decline: −5.04 (−6.80 to −3.28) vs −0.86 (−1.10 to −0.63) mL/min/1.73 m²/year; *P* < .001] and continued to have a steeper rate of decline over the year after the event [average eGFR decline: −3.83 (−5.30 to −2.35); *P* < .001] (*Table 2* and *Figure 4*).

**Table 2:** Estimated glomerular filtration rate trajectory before and after heart failure events in the eplerenone/placebo groups from the EPHESUS and EMPHASIS-HF trials

	Estimated rate of eGFR decline (mL/min/year)	Delta of estimated rate of eGFR decline in HF vs no HF event (mL/min/year)	*P*-value vs No HF event
Placebo			
No HF event	−0.86 (−1.10 to −0.63)		
> 1 year before HF event	−1.50 (−3.69 to 0.69)	−0.64 (−2.84 to 1.57)	.57
0–1 year before HF event	−5.04 (−6.80 to −3.28)	−4.18 (−5.95 to −2.40)	**<**.**0001**
0–1 year after HF event	−3.83 (−5.30 to −2.35)	−2.97 (−4.46 to −1.47)	.**0001**
> 1 year after HF event	0.31 (−1.78 to 2.40)	1.17 (−0.93 to 3.27)	.27
Eplerenone			
No HF event	−1.48 (−1.71 to −1.25)		
> 1 year before HF event	−1.15 (−3.10 to 0.80)	0.33 (−1.64 to 2.29)	.74
0–1 year before HF event	−4.66 (−6.41 to −2.91)	−3.18 (−4.94 to −1.41)	.**0004**
0–1 year after HF event	−2.95 (−4.63 to −1.27)	−1.47 (−3.17 to 0.23)	.089
> 1 year after HF event	−4.03 (−6.40 to −1.65)	−2.54 (−4.93 to −0.16)	.**037**

HF, heart failure; eGFR, estimated glomerular filtration rate; CI, confidence interval.

In the eplerenone group, patients with an HF event also had a steeper decline in eGFR during the year before the event, compared with those without an event [average eGFR decline: −4.66 (−6.41 to −2.91) vs −1.48 (−1.71 to −1.25) mL/min/1.73 m²/year; *P* < .001]. However, the rate of eGFR decline over the year after an HF event was not statistically different from that of patients without the event (*P* = .09) (*Table 2* and *Figure 4*).

## Discussion

Using data from two clinical trials (i.e. EPHESUS and EMPHASIS-HF) and a ‘real world’ study (BARCELONA cohort), we have shown that, among patients with HFrEF, declines in kidney function were notably accelerated in the year before a worsening HF event, with a slower rate of decline observed over the year after such events. Importantly, among patients receiving placebo, kidney function continued to decline relatively rapidly over the year after an HF event, whereas, among patients receiving eplerenone, the decline over the year after an HF event was comparable to the rate of decrease in patients without an event (*Structured Graphical Abstract*). Taken together, HF hospitalization impacted kidney function decline in patients with HFpEF.^9^ However, among patients with HFrEF, who are more susceptible to HF-related events, our findings suggest a paradigm shift, showing eGFR decline occurs not just weeks but up to a year before HF hospitalization. Targeting eGFR slopes, rather than relying solely on specific thresholds (e.g. 30 mL/min/1.73 m²), could encourage earlier detection of clinically meaningful kidney function decline and enable timely intervention.

Our findings align with those from the PARAGON-HF trial, which included 2306 patients with HFpEF (LVEF ≥45%), showing a rapid decline in eGFR during the year before and after an HF event, with a decrease of −1.5 mL/min/1.73 m²/year >1 year before, −2.8 mL/min/1.73 m²/year during 1 year before, −3.0 mL/min/1.73 m²/year during 1 year after, and −1.7 mL/min/1.73 m²/year >1 year after the events, compared with −1.7 mL/min/1.73 m² in those without the event.^9^ Additionally, eGFR trajectories have been shown to be associated with the risk of new-onset HF in the general communities.^15,16^ These findings provide novel insights into the intricate interplay between the heart and kidney in HF, highlighting that kidney function may progressively decline even in the medium term before HF events across a broad spectrum of LVEF.

Declines in eGFR are closely associated with underlying CV risk factors, CV diseases and disease severity,^17,18^ which have a dual impact on both heart and kidney, potentially fostering tissue fibrosis and disease progression.^19^ In the present analysis, patients experiencing HF events more often had concomitant CV conditions and a lower baseline eGFR compared with those not experiencing HF events, suggesting a greater likelihood of accelerated kidney function decline in these patients. This line of reasoning points to a shared underlying mechanism for the simultaneous progression of HF and kidney disease, and our study underscores this perspective by highlighting the dynamic nature of eGFR decline. Particularly noteworthy was our observation in the BARCELONA cohort, where eGFR was measured frequently during a relatively long follow-up period of 47 months. Here, our data showed that kidney function declined steeper both 1 year before (≈6 mL/min/1.73 m²/year) and after (≈3 mL/min/1.73 m²/year) HF events, compared with the ‘usual’ kidney function decline (1.5 mL/min/1.73 m²/year). Compared with the year before an HF event, the rate of kidney function declines after the event began to decelerate, eventually returning to the baseline level of kidney function decline (≈2 mL/min/1.73 m²/year). Therefore, these findings suggest that subclinical eGFR decline was related to the subsequent occurrence of HF events, possibly influenced by dynamic factors such as congestion.^20^

Furthermore, the observed deceleration in eGFR decline after one year may reflect the end of the ‘vulnerable phase’ that follows an HF event,^3,21^ possibly linked to partial recompensation and improved hemodynamics. By contrast, the steep decline prior to hospitalization is unlikely to be primarily driven by the initiation of new medications. In the clinical trial populations, most patients were already receiving optimized medical therapy at baseline, limiting the scope for further therapeutic intensification. While some treatment changes may have occurred in the real-world BARCELONA cohort, the consistency of the eGFR trajectory pattern across datasets supports the presence of a shared underlying physiological mechanism. However, future studies should explore in greater detail the role of treatment adjustments and their interaction with eGFR trajectories following HF hospitalization.

The observation that eGFR decline is steeper and more prolonged in patients with multiple HF events further supports the notion of a progressive and dynamic cardiorenal interaction. These results suggest that a persistently declining eGFR slope after a first hospitalization may predispose to repeated HF events and could serve as a critical warning signal to prompt earlier and more aggressive therapeutic intervention.

We performed several sensitivity analyses to assess the robustness of our findings. These included (i) excluding eGFR values within 2 weeks before and after the HF event to minimize the influence of acute, reversible fluctuations; (ii) defining exposure strictly as non-fatal HF hospitalization; and (iii) restricting the HF hospitalization analysis to patients who survived the entire follow-up, thereby mitigating the impact of competing risks and enhancing the reliability of post-event slope estimates. While confounding is not controlled at the time of the HF event, we observed consistent patterns across all models, suggesting that the associations are not artefacts of modelling strategy or driven by transient changes in kidney function during acute events. Nonetheless, several limitations of the data set must be acknowledged. Follow-up duration before and after the event varied across individuals, and post-event slopes may be affected by selective survival, particularly in patients with early death. Additionally, our modelling approach does not incorporate time-updated covariates, which may limit the ability to fully disentangle the intrinsic relationship between kidney function trajectories and outcomes. Future studies leveraging more complex longitudinal models including multivariable, time-updated frameworks could more precisely capture these dynamic interactions. Still, the consistency of results across all sensitivity analyses reinforces the plausibility and potential clinical relevance of the observed associations.

Renal congestion is a key factor affecting kidney function among patients with HF.^22^ Numerous preclinical and clinical studies have shown that increased central and renal venous pressures, along with maladaptive sodium and fluid overload, reduce renal blood flow, elevate renal interstitial pressures,^23,24^ and lead to declines in glomerular filtration rate as well as overall kidney function.^25–27^ Additionally, reduced renal blood flow may restrict the delivery of diuretics to the kidney, while declining glomerular filtration limits the tubular transport of sodium, resulting in diuretic resistance.^28^ Furthermore, worsening congestion may impair oral diuretic absorption due to gastrointestinal oedema or gut hypoperfusion, requiring diuretic dosage intensification and subsequently accelerating kidney function decline.^29^ In this study, the association between a steeper eGFR decline and NYHA class-based functional deterioration supports the hypothesis that worsening kidney function may reflect subclinical congestion. These data highlight that eGFR slope may serve as a more sensitive early warning signal than clinical status, which is often subjectively reported and less granular in routine practice. Furthermore, to explore the potential pathophysiological link between kidney function decline and HF hospitalization, we adjusted our models for NYHA class as a proxy for congestion. This led to a partial attenuation of the association between eGFR slope and HF events, particularly during the year prior to HF events, whereas the slope remained stable in patients without HF events. These findings suggest that clinical deterioration possibly due to congestion may partly mediate the relationship between kidney function decline and subsequent HF events. However, the persistent slope after adjustment for NYHA class suggests additional underlying mechanisms beyond NYHA class.

We observed that the rate of kidney function during 1 year after an HF event was steeper in the placebo group but less pronounced in the eplerenone group, compared with those without the event in both groups. Although MRA therapy is very effective in HFrEF, its initiation or up-titration is often limited due to impaired renal function.^30–34^ Our data, however, suggest that eplerenone helped preserve renal function even after the HF events, regardless of baseline kidney function severity. The non-steroidal MRA, finerenone, also has renal protective effects by decelerating eGFR decline in patients with diabetic kidney disease^35,36^ but has neutral effects on eGFR decline in those with HFpEF.^37^ The interactions between eGFR decline, HF events and MRA therapy warrant further prospective studies in the field of HF.

The main limitation of the present analysis was its *post hoc* nature, which means the findings should be regarded as hypothesis-generating. The data used for the analysis of eGFR trajectory, especially following HF events in the EPHESUS and EMPHASIS-HF trials, showed relatively moderate levels of granularity. This was particularly evident in the frequency of eGFR measurements available per patient, which may limit the precision of this analysis. Additionally, those with baseline eGFR <30 mL/min/1.73 m^2^ were excluded by the eligibility criteria of the EPHESUS and EMPHASIS-HF trials. Patients in the EPHESUS trial were not defined as an HF population; however, > 90% of patients had symptomatic HF and experienced more frequent HF events than MI or stroke, suggesting that this population was similar to those with HFrEF. Additionally, as a sensitivity analysis, we observed similar trajectories of kidney function among patients in the EMPHASIS-HF trial only. Although we explored the potential mediating role of congestion using NYHA class adjustment, a formal mediation analysis could not be performed due to the complexity of slope modelling. Moreover, NYHA class may not fully capture subclinical congestion. Future work incorporating objective haemodynamic or biomarker data is needed to better understand the mechanisms linking eGFR decline to HF events. Natriuretic peptide and Kansas City Cardiomyopathy Questionnaire data were not available with sufficient consistency across the datasets to allow meaningful longitudinal analysis. This limits our ability to directly correlate eGFR trajectories with further measures of congestion and should be addressed in future studies. Furthermore, since serum creatinine was measured only in patients who survived hospitalization, our analysis was subject to a degree of survival bias. Patient characteristics at the time of the HF event were not available, as data were collected during predefined visits rather than at the time of clinical events.

Sodium-glucose co-transporter 2 inhibitors and sacubitril/valsartan have been shown to slow the progression of kidney function decline in patients with HFrEF.^38,39^ However, given the timeframe of the EPHESUS and EMPHASIS-HF trials and the limited uptake of these therapies in the earlier years of the BARCELONA cohort, we were not able to assess their specific effects on eGFR trajectories around HF events. Future analyses in contemporary trial populations are needed to determine how the association between kidney function decline and HF hospitalization may be influenced by these newer therapies, and to what extent their benefits are mediated through renal mechanisms.

## Conclusions

In patients with HFrEF, kidney function decline may precede a HF hospitalization or death by up to 1 year. These data suggest that incorporating eGFR slopes into clinical decision-making could help identify patients at higher risk for HF hospitalizations in the mid-term, potentially enabling early intervention.

## Supplementary Material

ehaf457_Supplementary_Data

## Declarations

### Data Availability

Data can be made available upon reasonable request to the steering committees of the studies.

### Pre-registered Clinical Trial Number

EMPHASIS-HF: ClinicalTrials.gov Identifier NCT00232180 EPHESUS: not registered on ClinicalTrials.gov (study initiated before 2000) BARCELONA cohort: observational registry, not applicable.
