# Supplementary material for: Kidney function trajectories before and after hospitalization for heart failure with reduced ejection fraction
Source: Eur Heart J. 2025 Jul 30;46(43):4583–93. doi: 10.1093/eurheartj/ehaf457 (PMC12614968; doi:10.1093/eurheartj/ehaf457)

**Supplemental materials**

**Supplemental table 1. eGFR trajectory before and after HF events in the EMPHASIS-HF trial**

|  | Estimated rate of eGFR decline (ml/min/year) | P-value vs.  No HF event |
| --- | --- | --- |
| No HF event | -0.85 (-1.10 to -0.61) |  |
| Before HF event |  |  |
| Entire period | -2.51 (-3.49 to -1.53) | **<0.0001** |
| >1 year before HF event | -2.01 (-3.58 to -0.43) | **0.013** |
| 0-1 year before HF event | -3.42 (-5.86 to -0.98) | **0.006** |
| After HF event |  |  |
| Entire period |  |  |
| 0-1 year after HF event | -3.43 (-6.68 to -0.18) | **0.039** |
| >1 year after HF event | -1.64 (-3.63 to 0.35) | 0.11 |

HF, heart failure; eGFR, estimated glomerular filtration rate; CI, confidence interval.

**Supplemental table 2. eGFR trajectory before and after HF events from EPHESUS and EMPHASIS-HF trials: model adjusted for NYHA class**

| **No HF event** | **Before HF event** | | |
| --- | --- | --- | --- |
| **Estimated rate of eGFR decline/yr**  **(95% CI)** | **Timing** | **Estimated rate of**  **eGFR decline/yr**  **(95% CI)** | **P-value vs. No HF event** |
| -1.09 (-1.27 to -0.91) | **Entire period** | -2.52  (-3.26 to -1.78) | 0.0002 |
|  | **>1 year before** | -1.47  (-2.94 to -0.00) | 0.61 |
|  | **0-1 year before** | -3.31  (-4.66 to -1.96) | 0.001 |

HF, heart failure; eGFR, estimated glomerular filtration rate; CI, confidence interval.

**Supplemental table 3. NYHA trajectory before and after HF events from EPHESUS and EMPHASIS-HF trials, with tertiles of slope eGFR calculated in the year before HF event for patients with HF event**

| **No HF event** | **Before HF event** | | | **After HF event** | | |
| --- | --- | --- | --- | --- | --- | --- |
| **Estimated rate of eGFR decline/yr**  **(95% CI)** | **Timing** | **Estimated rate of eGFR decline/yr**  **(95% CI)** | **P-value vs. No HF event** | **Timing** | **Estimated rate of eGFR decline/yr**  **(95% CI)** | **P-value vs. No HF event** |
| -0.06  (-0.06 to -0.05) | **Entire period** |  |  | **Entire period** |  |  |
|  | **Tertile 1** | 0.39 (0.32 to 0.46) | <0.0001 | **Tertile 1** | -0.16 (-0.19 to -0.12) | <0.0001 |
|  | **Tertile 2** | 0.13 (0.10 to 0.16) | <0.0001 | **Tertile 2** | 0.01 (-0.02 to 0.05) | <0.0001 |
|  | **Tertile 3** | 0.11 (0.07 to 0.14) | <0.0001 | **Tertile 3** | 0.02 (-0.02 to 0.05) | <0.0001 |
|  | **>1 year before** |  |  | **0-1 year after** |  |  |
|  | **Tertile 1** | -0.08 (-0.25 to 0.09) | 0.79 | **Tertile 1** | -0.30 (-0.36 to -0.24) | <0.0001 |
|  | **Tertile 2** | -0.05 (-0.10 to 0.00) | 0.70 | **Tertile 2** | -0.08 (-0.14 to -0.02) | 0.58 |
|  | **Tertile 3** | -0.11 (-0.17 to -0.04) | 0.13 | **Tertile 3** | -0.06 (-0.12 to 0.00) | 0.96 |
|  | **0-1 year before** |  |  | **>1 year after** |  |  |
|  | **Tertile 1** | 0.62 (0.52 to 0.71) | <0.0001 | **Tertile 1** | 0.04 (-0.05 to 0.13) | 0.032 |
|  | **Tertile 2** | 0.31 (0.26 to 0.36) | <0.0001 | **Tertile 2** | 0.04 (-0.03 to 0.11) | 0.006 |
|  | **Tertile 3** | 0.34 (0.27 to 0.41) | <0.0001 | **Tertile 3** | 0.06 (-0.03 to 0.15) | 0.012 |

HF, heart failure; eGFR, estimated glomerular filtration rate; CI, confidence interval.

HF event groups were stratified with three slope-based categories: tertile 1 (steep eGFR decline, <-17.5 mL/min/1.73m²/year), tertile 2 (mild decline or stable eGFR, -17.5 to 1.9 mL/min/1.73m²/year), and tertile 3 (eGFR increase, >1.9 mL/min/1.73m²/year). Slopes were calculated using the last measurement >1 year prior, all values within 1 year before, and the first value after the HF event.

**Supplemental table 4. eGFR trajectory after one or multiple HF events from EPHESUS and EMPHASIS-HF trials, and BARCELONA cohort**

| **EPHESUS/EMPHASIS-HF** | | |
| --- | --- | --- |
| **Timing** | **Beta (95% CI)** | **P-value vs. No HF event** |
| **Only one HF event** |  |  |
| **Entire period** | -2.03 (-2.88 to -1.17) | 0.056 |
| **0-1 year after** | -2.86 (-4.23 to -1.48) | 0.017 |
| **>1 year after** | 0.17 (-1.90 to 2.23) | 0.20 |
| **Multiple HF events** |  |  |
| **Entire period** | -4.32 (-5.33 to -3.32) | <0.0001 |
| **0-1 year after** | -4.37 (-6.05 to -2.69) | 0.0002 |
| **>1 year after** | -3.71 (-6.11 to -1.32) | 0.039 |
| **BARCELONA cohort** | | |
| **Timing** | **Beta (95% CI)** | **P-value vs. No HF event** |
| **Only one HF event** |  |  |
| **Entire period** | -1.82 (-2.08 to -1.55) | 0.0007 |
| **0-1 year after** | -1.26 (-2.70 to 0.18) | 0.90 |
| **>1 year after** | -1.62 (-1.97 to -1.28) | 0.12 |
| **Multiple HF events** |  |  |
| **Entire period** | -2.07 (-2.21 to -1.94) | <0.0001 |
| **0-1 year after** | -3.73 (-4.72 to -2.74) | <0.0001 |
| **>1 year after** | -1.65 (-1.82 to -1.48) | 0.0008 |

**Supplemental table 5. eGFR decline modeled in three patient groups: (i) alive without HF event, (ii) death during follow-up without prior HF hospitalization, (iii) non-fatal HF hospitalization as first event (irrespective of subsequent mortality)**

| **EPHESUS/EMPHASIS-HF** | | | | | | | | | |
| --- | --- | --- | --- | --- | --- | --- | --- | --- | --- |
| **Alive without non-fatal HF event** | **Before non-fatal HF event/death** | | | **After non-fatal HF event** | | | | | |
| **Beta (95% CI)** | **Timing** | **Beta (95% CI)** | **P-value vs. Alive without non-fatal HF event** | **Timing** | **Beta (95% CI)** | | **P-value vs. Alive without non-fatal HF event** | | |
| -1.13 (-1.30 to -0.96) | **Before non-fatal HF event** |  |  | **After non-fatal HF event** |  | |  | | |
|  | **Entire period** | -3.38 (-4.10 to -2.66) | <0.0001 | **Entire period** | -3.00 (-3.66 to -2.33) | | <0.0001 | | |
|  | **>1 year before** | -1.44 (-2.93 to 0.05) | 0.69 | **0-1 year after** | -3.51 (-4.62 to -2.40) | | <0.0001 | | |
|  | **0-1 year before** | -4.64 (-5.90 to -3.38) | <0.0001 | **>1 year after** | -1.55 (-3.12 to 0.02) | | 0.60 | | |
|  | **Before death** |  |  |  |  | |  | | |
|  | **Entire period** | -2.04 (-3.09 to -1.00) | 0.091 |  |  | |  | | |
|  | **>1 year before** | 0.91 (-0.99 to 2.81) | 0.036 |  |  | |  | | |
|  | **0-1 year before** | -4.91 (-6.77 to -3.05) | <0.0001 |  |  | |  | | |
| **BARCELONA cohort** | | | | | | | | | |
| **Alive without non-fatal HF event** | **Before non-fatal HF event/death** | | | **After non-fatal HF event** | | | | |  |
| **Beta (95% CI)** | **Timing** | **Beta (95% CI)** | **P-value vs. Alive without non-fatal HF event** | **Timing** | | **Beta (95% CI)** | | **P-value vs. Alive without non-fatal HF event** | |
| -1.29 (-1.36 to -1.23) | **Before non-fatal HF event** |  |  | **After non-fatal HF event** | |  | |  | |
|  | **Entire period** | -2.50 (-2.60 to -2.41) | <0.0001 | **Entire period** | | -2.02 (-2.14 to -1.89) | | <0.0001 | |
|  | **>1 year before** | -2.14 (-2.25 to -2.02) | <0.0001 | **0-1 year after** | | -3.03 (-3.94 to -2.13) | | 0.0002 | |
|  | **0-1 year before** | -5.75 (-6.60 to -4.91) | <0.0001 | **>1 year after** | | -1.66 (-1.81 to -1.50) | | <0.0001 | |
|  | **Before death** |  |  |  | |  | |  | |
|  | **Entire period** | -1.48 (-1.59 to -1.37) | 0.005 |  | |  | |  | |
|  | **>1 year before** | -1.40 (-1.52 to -1.28) | 0.12 |  | |  | |  | |
|  | **0-1 year before** | -4.56 (-6.08 to -3.04) | <0.0001 |  | |  | |  | |

**Supplemental table 6. eGFR decline modeled in three patient groups: (i) alive without HF event, (ii) death during follow-up with or without prior HF hospitalization, (iii) non-fatal HF hospitalization as first event without subsequent mortality**

|  | **EPHESUS/EMPHASIS-HF** | | | |  | | | | |
| --- | --- | --- | --- | --- | --- | --- | --- | --- | --- |
| **Alive without non-fatal HF event** | **Before non-fatal HF event/death** | | | | **After non-fatal HF event** | | | | |
| **Beta (95% CI)** | **Timing** | **Beta (95% CI)** | **P-value vs. Alive without non-fatal HF event** | | **Timing** | **Beta (95% CI)** | | **P-value vs. Alive without non-fatal HF event** | |
| -1.13 (-1.30 to -0.96) | **Before non-fatal HF event** |  |  | | **After non-fatal HF event** |  | |  | |
|  | **Entire period** | -3.18 (-4.01 to -2.35) | <0.0001 | | **Entire period** | -2.62 (-3.33 to -1.92) | | <0.0001 | |
|  | **>1 year before** | -1.17 (-2.88 to 0.54) | 0.96 | | **0-1 year after** | -3.04 (-4.24 to -1.84) | | 0.002 | |
|  | **0-1 year before** | -4.49 (-5.94 to -3.04) | <0.0001 | | **>1 year after** | -1.36 (-3.02 to 0.30) | | 0.79 | |
|  | **Before death** |  |  | |  |  | |  | |
|  | **Entire period** | -3.46 (-4.22 to -2.71) | <0.0001 | |  |  | |  | |
|  | **>1 year before** | -1.10 (-2.40 to 0.21) | 0.96 | |  |  | |  | |
|  | **0-1 year before** | -6.18 (-7.62 to -4.74) | <0.0001 | |  |  | |  | |
|  | **BARCELONA cohort** | | | |  | | | | |
| **Alive without non-fatal HF event** | **Before non-fatal HF event/death** | | | | **After non-fatal HF event** | | | | |
| **Beta (95% CI)** | **Timing** | **Beta (95% CI)** | | **P-value vs. Alive without non-fatal HF event** | **Timing** | | **Beta (95% CI)** | | **P-value vs. Alive without non-fatal HF event** |
| -1.29 (-1.36 to -1.23) | **Before non-fatal HF event** |  | |  | **After non-fatal HF event** | |  | |  |
|  | **Entire period** | -2.20 (-2.37 to -2.03) | | <0.0001 | **Entire period** | | -1.58 (-1.74 to -1.41) | | 0.002 |
|  | **>1 year before** | -1.70 (-1.92 to -1.48) | | 0.0006 | **0-1 year after** | | -2.11 (-3.68 to -0.54) | | 0.31 |
|  | **0-1 year before** | -6.08 (-7.64 to -4.53) | | <0.0001 | **>1 year after** | | -1.32 (-1.52 to -1.12) | | 0.80 |
|  | **Before death** |  | |  |  | |  | |  |
|  | **Entire period** | -2.20 (-2.26 to -2.13) | | <0.0001 |  | |  | |  |
|  | **>1 year before** | -2.07 (-2.15 to -2.00) | | <0.0001 |  | |  | |  |
|  | **0-1 year before** | -6.64 (-7.61 to -5.67) | | <0.0001 |  | |  | |  |

# **Supplemental table 7: eGFR slopes in a sensitivity analysis excluding eGFR measurements collected within 2 weeks before or after HF event**

| **EPHESUS/EMPHASIS-HF** | | | | | | | | |
| --- | --- | --- | --- | --- | --- | --- | --- | --- |
| **No HF event** | **Before HF event** | | | | | **After HF event** | | |
| **Beta (95% CI)** | **Timing** | | **Beta (95% CI)** | | **P-value vs. No HF event** | **Timing** | **Beta (95% CI)** | **P-value vs. No HF event** |
| -1.11 (-1.29 to -0.93) | **Entire period** | | -3.20 (-3.94 to -2.46) | | <0.0001 | **Entire period** | -3.14 (-3.83 to -2.45) | <0.0001 |
|  | **>1 year before** | | -1.53 (-3.00 to -0.06) | | 0.58 | **0-1 year after** | -3.63 (-4.82 to -2.44) | <0.0001 |
|  | **0-1 year before** | | -4.34 (-5.68 to -3.01) | | <0.0001 | **>1 year after** | -1.75 (-3.34 to -0.17) | 0.43 |
| **BARCELONA cohort** | | | | | | | | |
| **No HF event** | | **Before HF event** | | | | **After HF event** | | |
| **Beta (95% CI)** | | **Timing** | | **Beta (95% CI)** | **P-value vs. No HF event** | **Timing** | **Beta (95% CI)** | **P-value vs. No HF event** |
| -1.34 (-1.40 to -1.27) | | **Entire period** | | -2.47 (-2.57 to -2.38) | <0.0001 | **Entire period** | -2.04 (-2.16 to -1.91) | <0.0001 |
|  | | **>1 year before** | | -2.12 (-2.24 to -2.01) | <0.0001 | **0-1 year after** | -3.07 (-4.03 to -2.11) | 0.0004 |
|  | | **0-1 year before** | | -5.61 (-6.51 to -4.71) | <0.0001 | **>1 year after** | -1.68 (-1.83 to -1.52) | <0.0001 |

**Supplemental figure 1: Trajectory of eGFR before and after heart failure events modeled using piecewise linear regression**


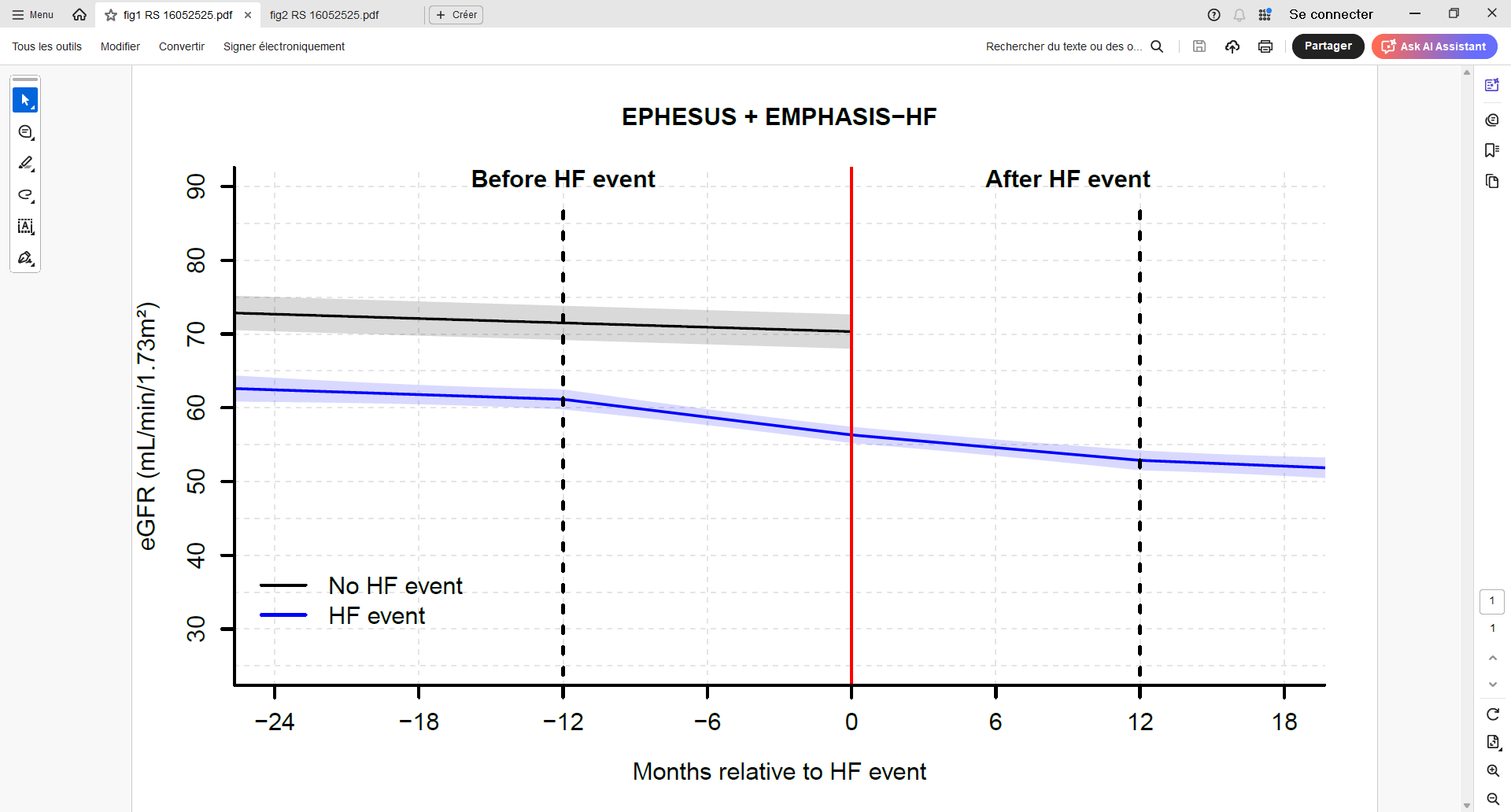


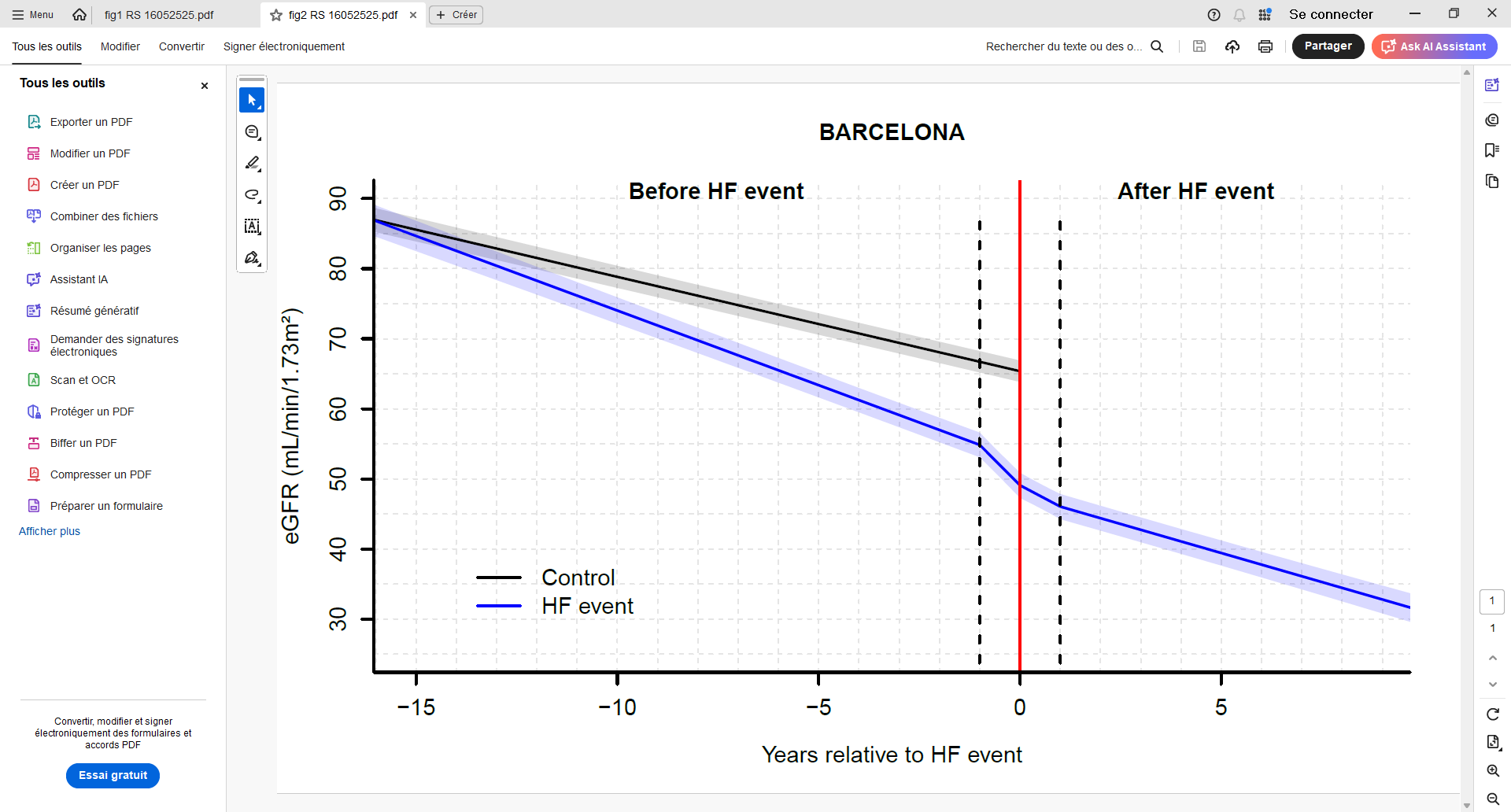


**Supplemental figure 2. eGFR decline modeled in three patient groups: (i) alive without HF event, (ii) death during follow-up without prior HF hospitalization, (iii) non-fatal HF hospitalization as first event (irrespective of subsequent mortality)**

**
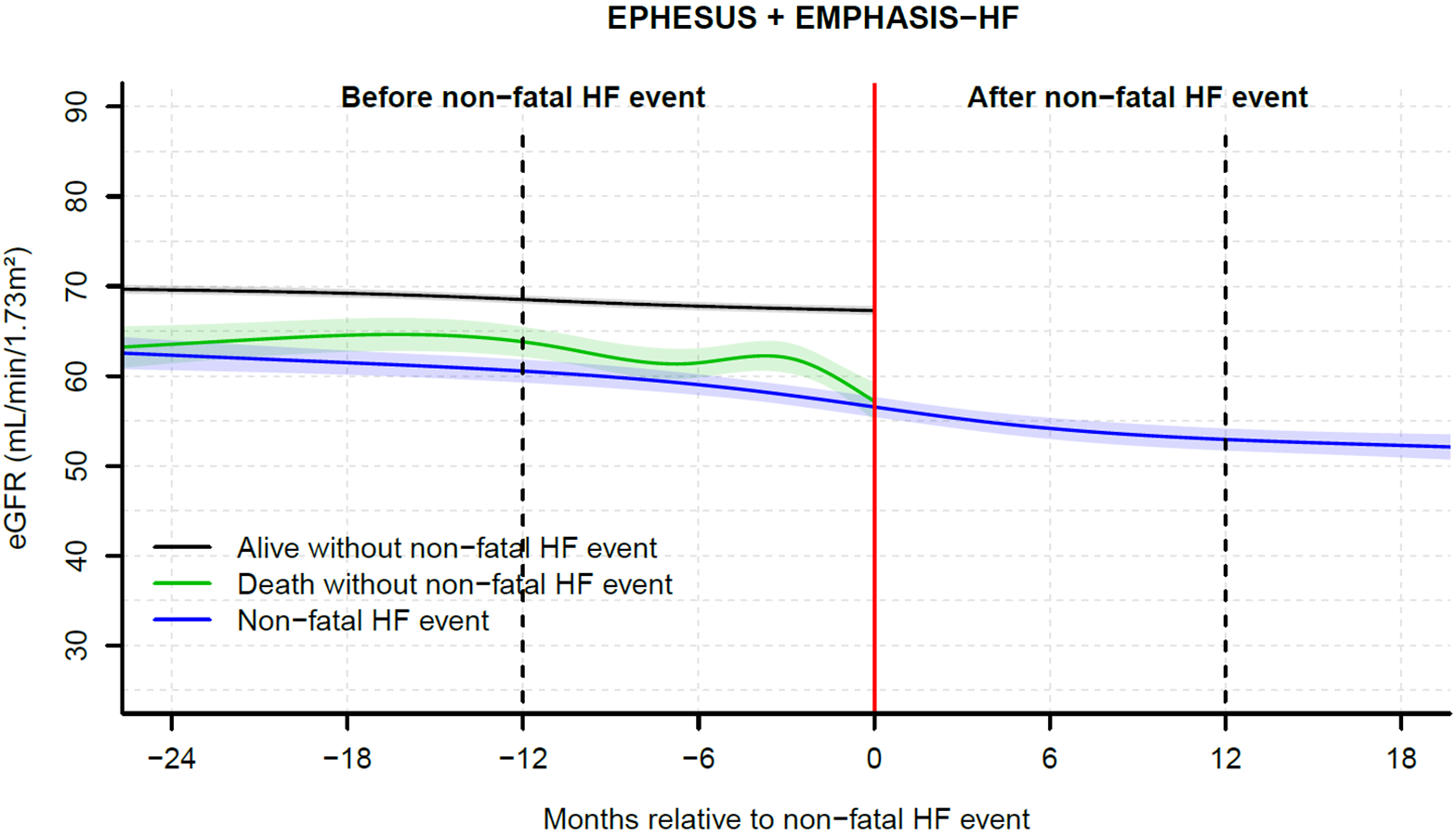
**


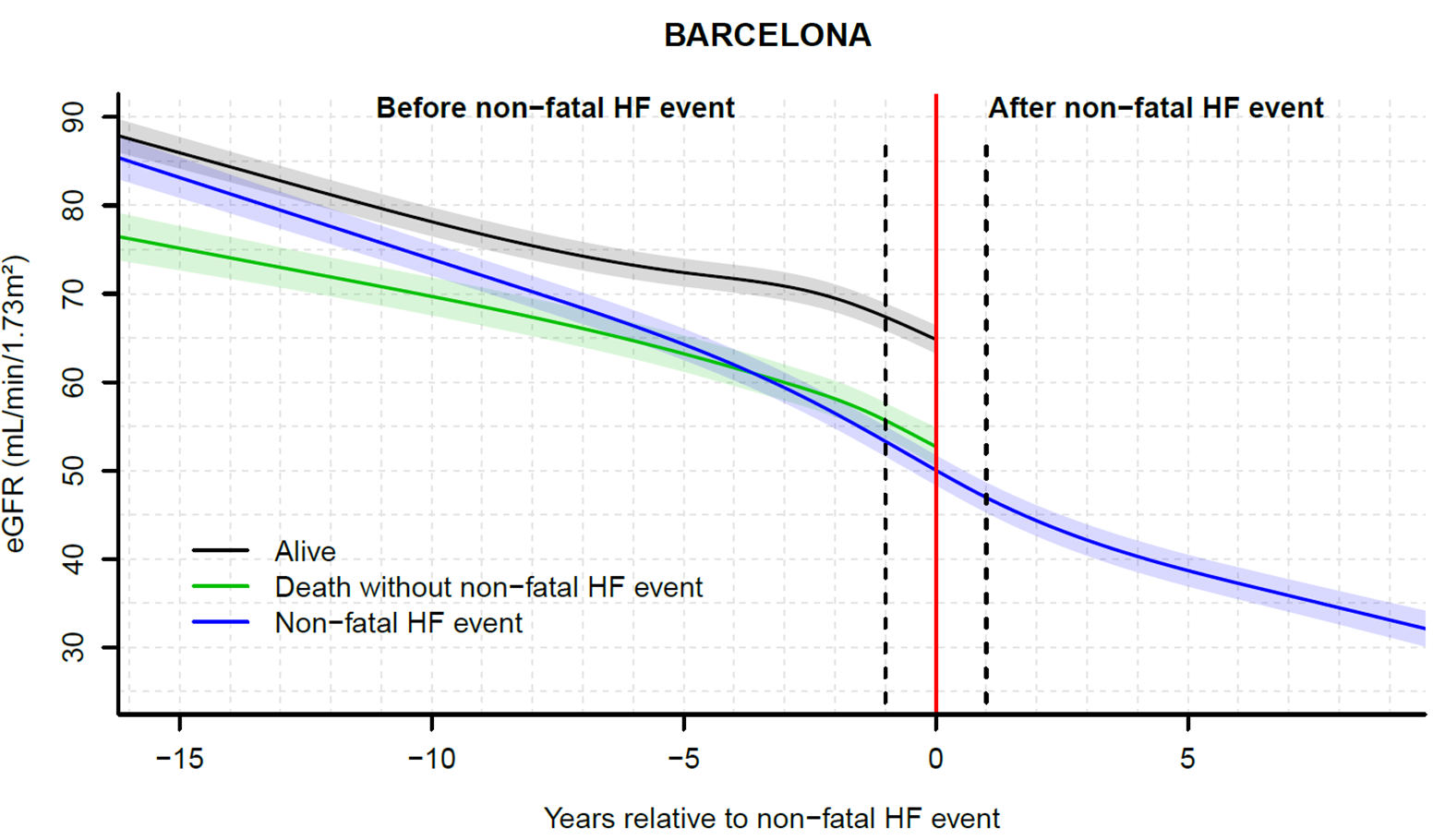

Supplement: ehaf457_Supplementary_Data [file ehaf457_supplementary_data.docx]
